# Supplementary material for: Dynamic inter-domain transformations mediate the allosteric regulation of human 5, 10-methylenetetrahydrofolate reductase
Source: Nat Commun. 2024 Apr 15;15:3248. doi: 10.1038/s41467-024-47174-y (PMC11018872; doi:10.1038/s41467-024-47174-y)
Supplement: Supplementary file 1 — Supplementary Information [file 41467_2024_47174_MOESM1_ESM.pdf]

Supplementary Information

Dynamic inter-domain transformations mediate the allosteric regulation of human 5, 10-methylenetetrahydrofolate reductase

Linnea K. M. Blomgren[1], Melanie Huber[1], Sabrina R. Mackinnon[2], Céline Bürer[1], Arnaud Baslé[2], Wyatt W. Yue[2,3] ✉, D. Sean Froese[1] ✉, Thomas J. McCorvie[2] ✉

[1]Division of Metabolism and Children's Research Center, University Children's Hospital Zürich, University of Zürich, Zürich, Switzerland

[2]Biosciences Institute, The Medical School, Newcastle University, Newcastle upon Tyne, NE2 4HH, UK

[3]Centre for Medicines Discovery, Nuffield Department of Clinical Medicine, University of Oxford, Oxford, OX3 7DQ, UK

✉e-mail: Wyatt.Yue@newcastle.ac.uk; Sean.Froese@kispi.uzh.ch; Thomas.McCorvie@newcastle.ac.uk

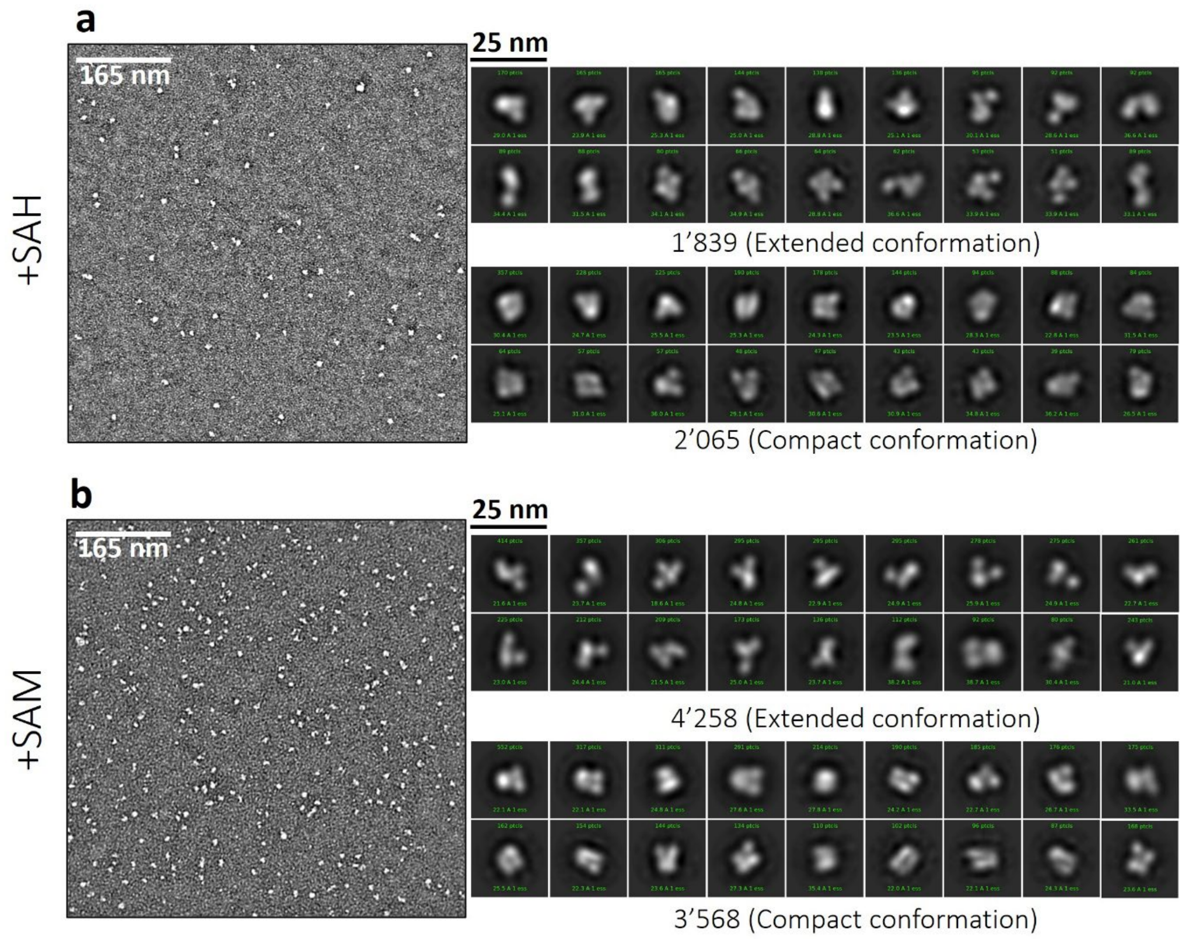

Supplementary Fig. 1 | Negative stain. Representative micrographs and 2D classes for **a**, $MTHFR_{FL}$ incubated with 2.6 mM SAH and **b**, $MTHFR_{FL}$ incubated with 5 mM SAM. Both samples exhibit two conformations: one more extended conformation (upper rows) resembling the structure of $MTHFR_{trunc}^{SAH}$ (PDB:6fcx[1]) and one more compact conformation (lower rows) likely to represent inhibited MTHFR.

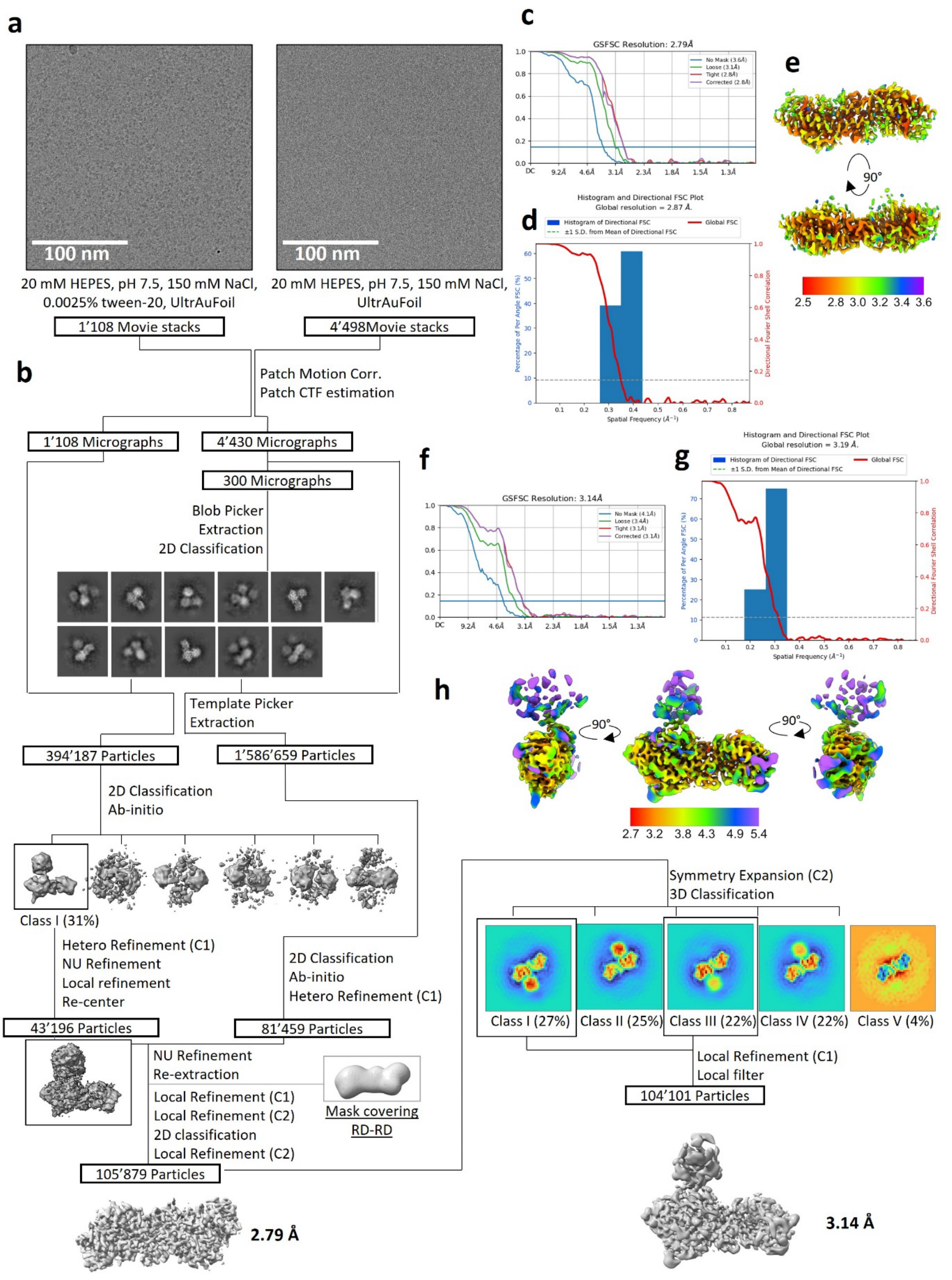

Supplementary Fig. 2 | Cryo-EM image processing workflow of MTHFR$_{FL}$^{SAH}. a, Representative micrographs from two movie stacks, of 1108 and 4498 micrographs respectively, collected with different grid conditions. **B**, Process flow chart for pooled data sets from which two maps for MTHFR$_{FL}$^{SAH} were reconstructed: One symmetric map MTHFR$_{FL}$$^{SAH\ (symm)}$ capturing the two regulatory

domains at 2.79 Å with corresponding **c**, FSC curve **d**, 3D-FSC plot showing the angular distribution and **e**, local resolution distribution. One asymmetric map $MTHFR_{FL}^{SAH\ (asymm)}$ capturing the two regulatory domains and low-resolution volume, due to high flexibility, representing one catalytic domain and linker region at average resolution of 3.14 Å, with corresponding **f**, FSC curve **g**, 3D-FSC plot showing the angular distribution and **h**, local resolution distribution.

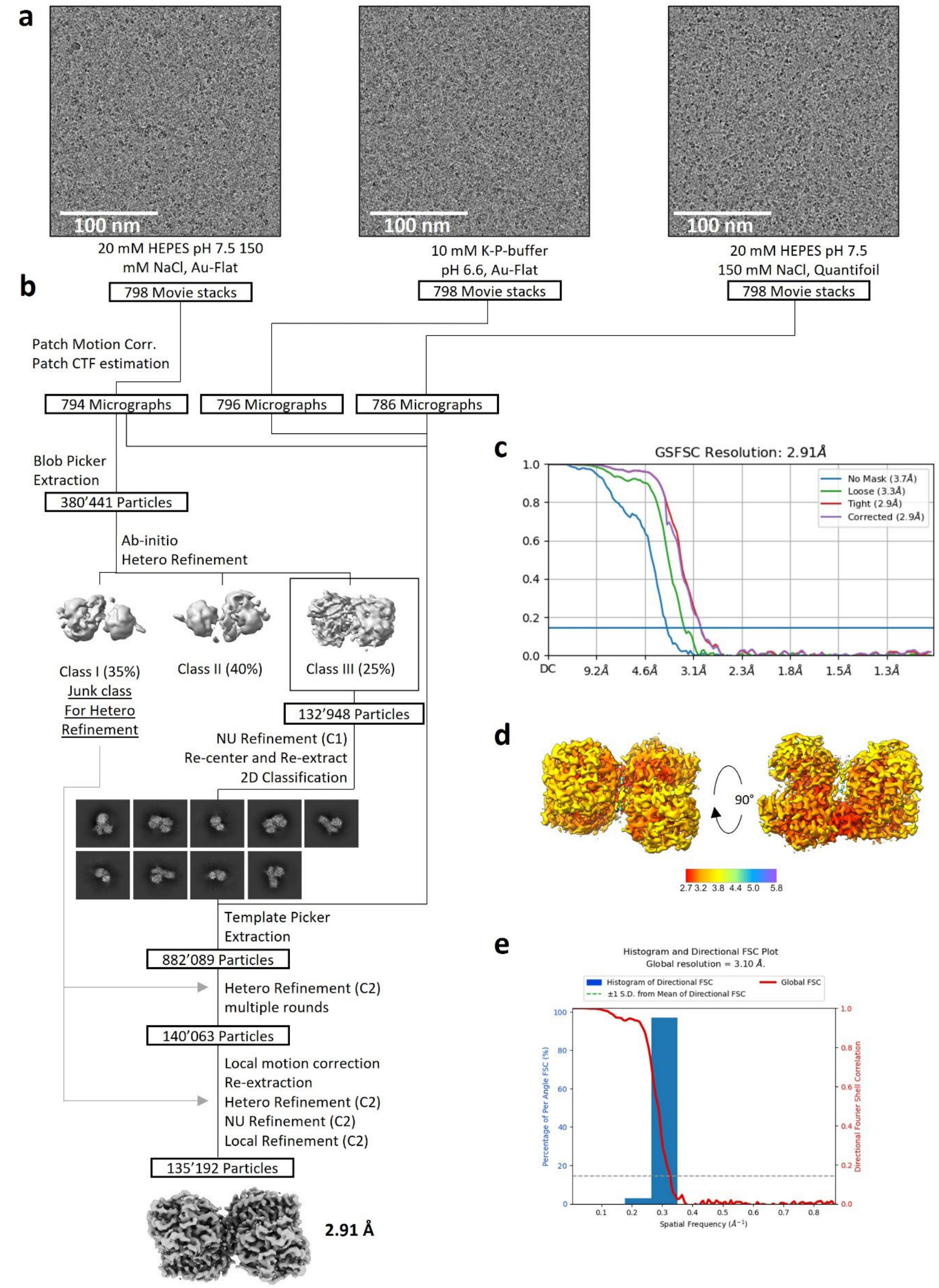

Supplementary Fig. 3 | Cryo-EM image processing workflow of MTHFR$_{FL}$^{SAM}. A, Representative micrographs from three movie stacks, each of 798 micrographs, collected with different grid

conditions. **b**, Process flow chart for pooled data sets from which one map for $MTHFR_{FL}^{SAM}$ at 2.91 Å was reconstructed with corresponding **c**, FSC curve **d**, local resolution distribution. and **e**, 3D-FSC plot showing the angular distribution.

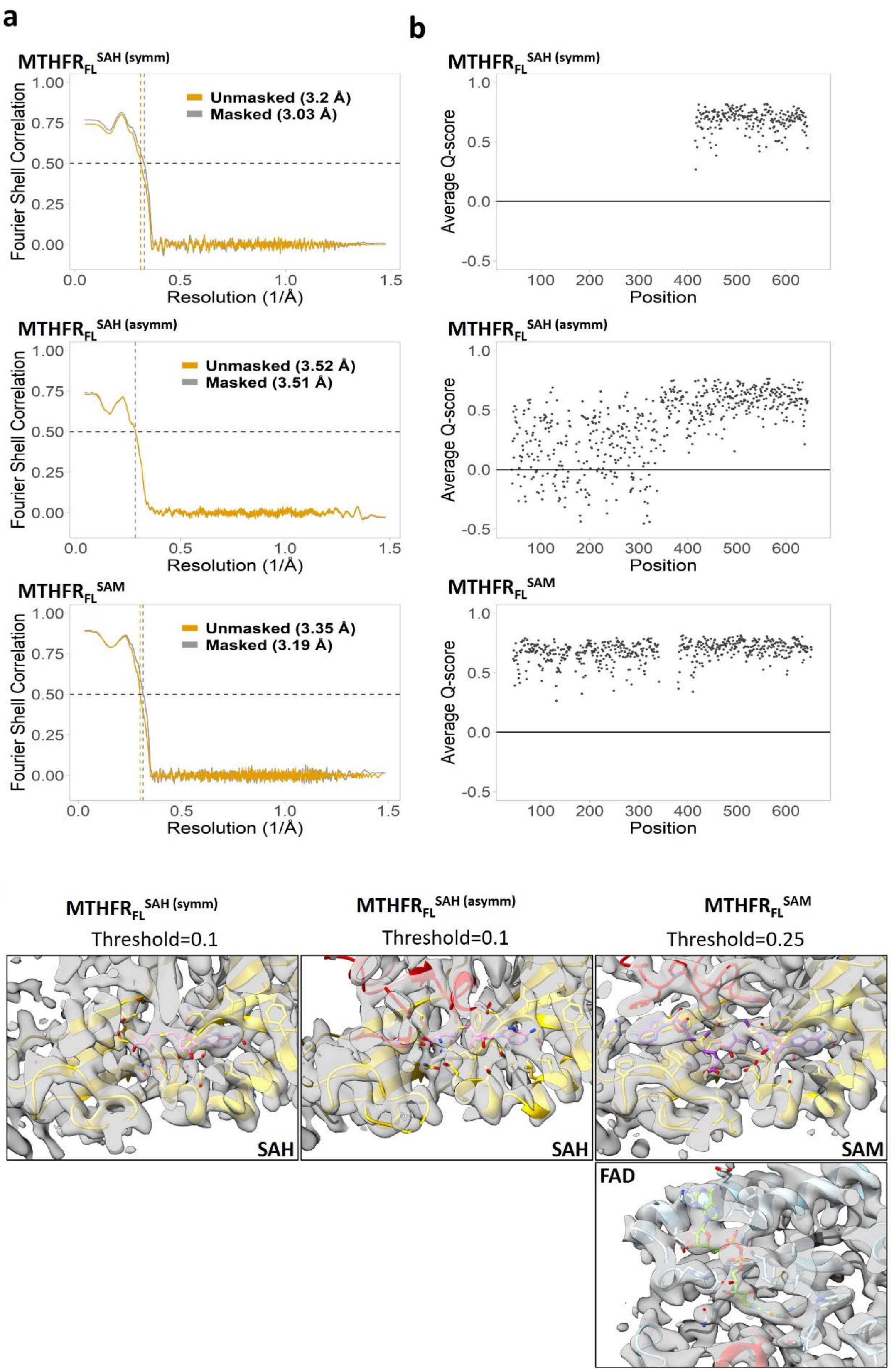

Supplementary Fig. 4 | Map and model quality of the MTHFR structures. Comparing models with respective final map: $MTHFR_{FL}^{SAH\ (symm)}$ with sharp map, $MTHFR_{FL}^{SAH\ (asymm)}$ with locally filtered map, and $MTHFR_{FL}^{SAM}$ with sharp map. **a**, Map to model FSC curves of the A-chains in as determined by Phenix validation[2] with printed resolution at FSC=0.5. **b**, Average Q-score[3] for each amino acid position in the

A-chains. The low scores for residues 48 to 338 of $MTHFR_{FL}^{SAH\ (asymm)}$ are due to the low resolution of this region corresponding to the flexible CD. **c,** Representative images of the models fitted within their respective map at specified threshold, showing A-chain ligands SAH, SAM, FAD and residues within a distance of 5Å as stick figures. Colors of the cartoon correspond to catalytic domain (blue), linker region (red) and regulatory domain (yellow).

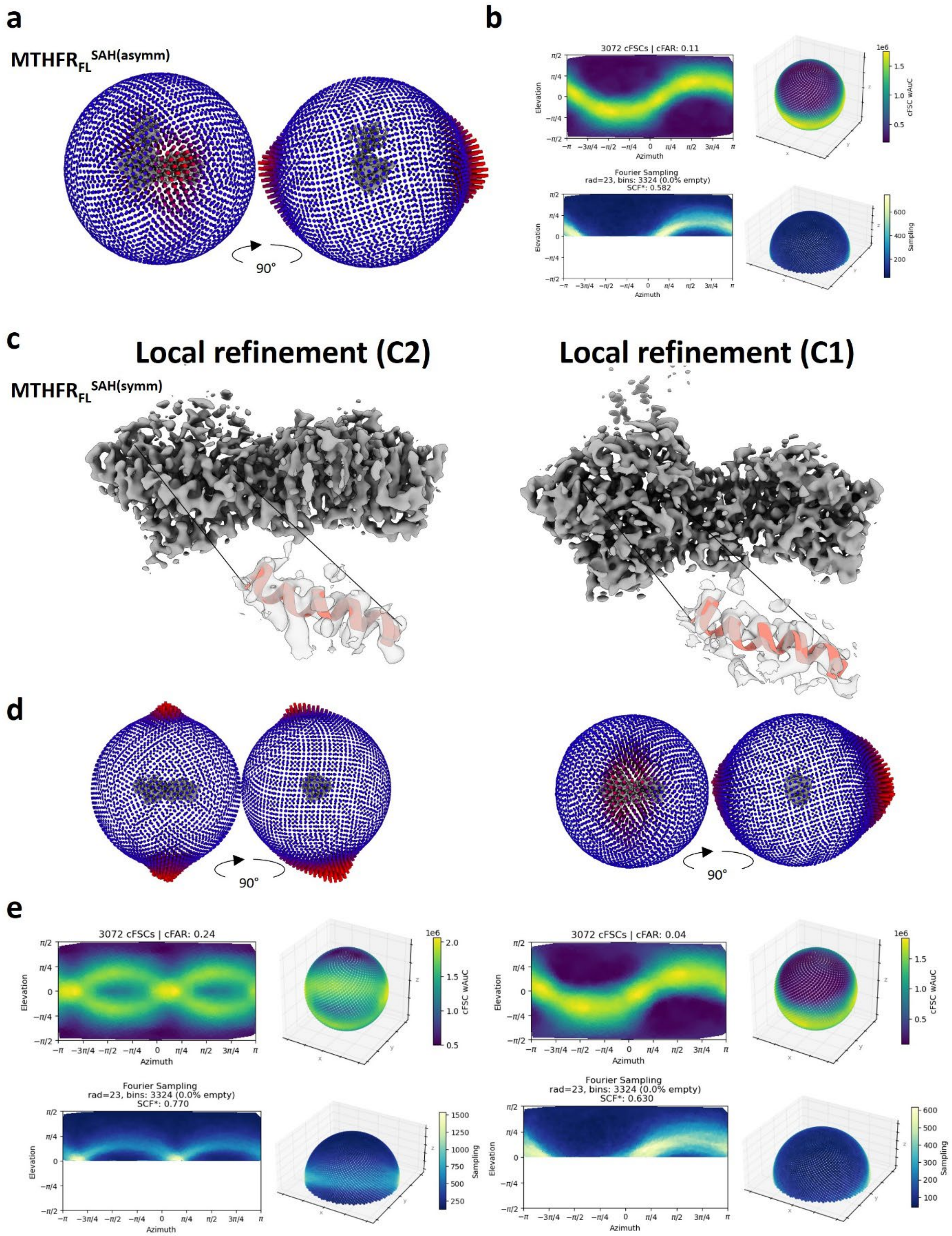

Supplementary Fig. 5 | Evaluation of C1 and C2 symmetry of MTHFR$_{FL}^{SAH}$ processing. a, 3D viewing direction distributions and **b**, cFAR and SCF* values for final map for MTHFR$_{FL}^{SAH(asymm)}$. Masked local refinement of the central regulatory domain dimer of the MTHFR SAH bound sample with C2 (deposited map MTHFR$_{FL}^{SAH(symm)}$) and without C2 symmetry and using the same mask. **c**, Resulting sharpened maps of masked local refinements of the RD dimer with C2 symmetry, left, and no symmetry applied (C1), right. An alpha-helix representing residues 576-595 is shown fitted in the

density for both maps. It is clear from visual inspection that the C2 symmetric map has secondary structure features whereas the locally refined map without symmetry does not. **d**, The corresponding 3D viewing direction distributions and **e**, cFAR and SFC* plots for the C2 symmetric, left, and C1, right, locally refined maps. An improvement in both cFAR and SFC* values are apparent when C2 symmetry is applied however the low cFAR of 0.24 indicates effects from orientation bias remain in the C2 symmetric map.

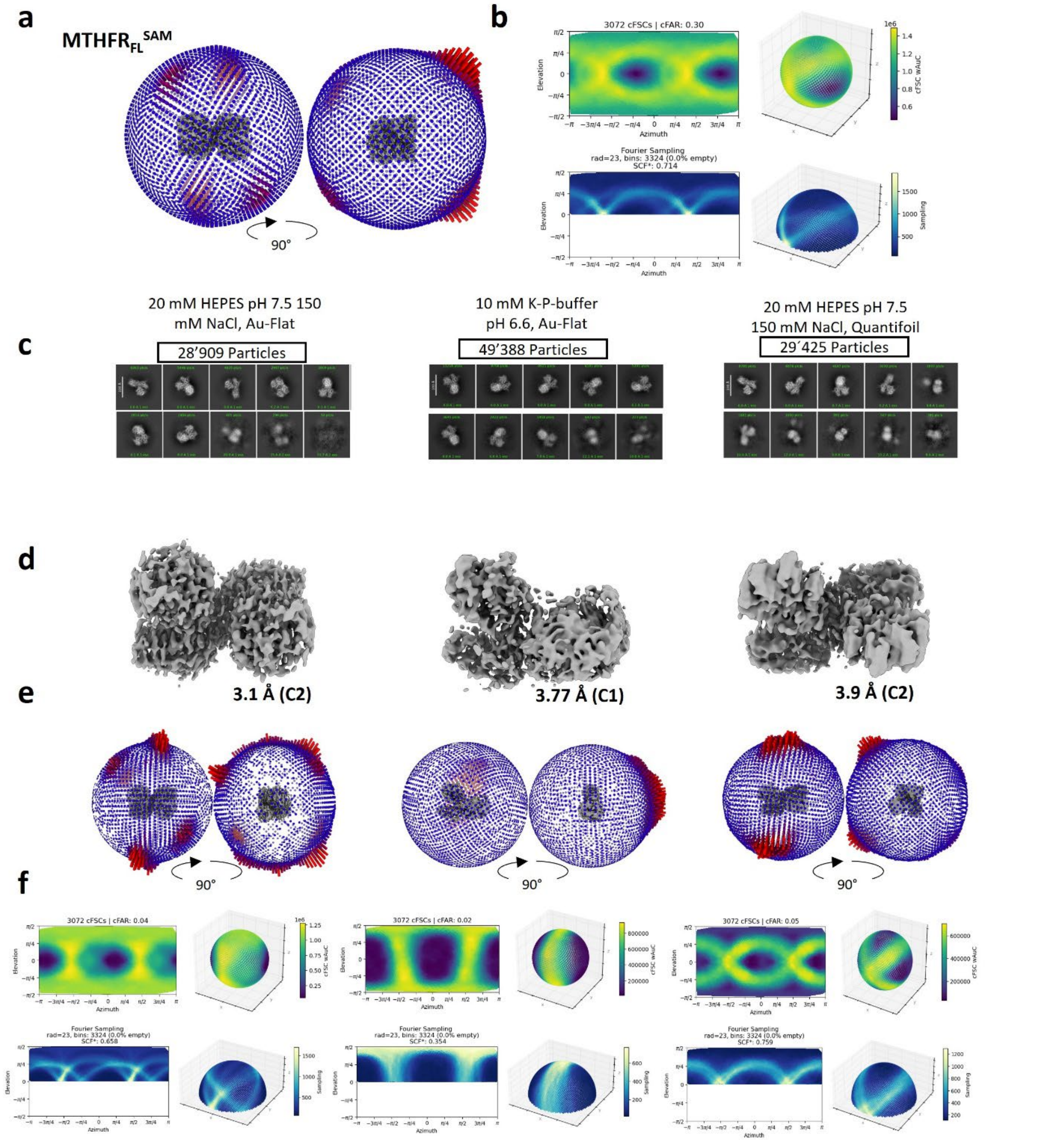

Supplementary Fig. 6 | Evaluation of the effect of grid conditions on orientation bias. **a**, 3D viewing direction distributions and **b**, cFAR and SCF* values for final map for $MTHFR_{FL}^{SAM}$. Respective grid conditions pooled to generate $MTHFR_{FL}^{SAM}$ processed individually from template picker job in Supplementary figure 3 with corresponding **c**, 2D classification job, followed by Ab-initio (not shown) and **d**, NU refinement with applied C1 symmetry, and when applicable C2 symmetry. Due to orientation bias grid condition “10 mM K-P-buffer pH 6.6, Au-Flat“ was not able to resolve the C2 symmetry. **e**, 3D viewing direction distributions and **f**, cFAR and SCF* values.

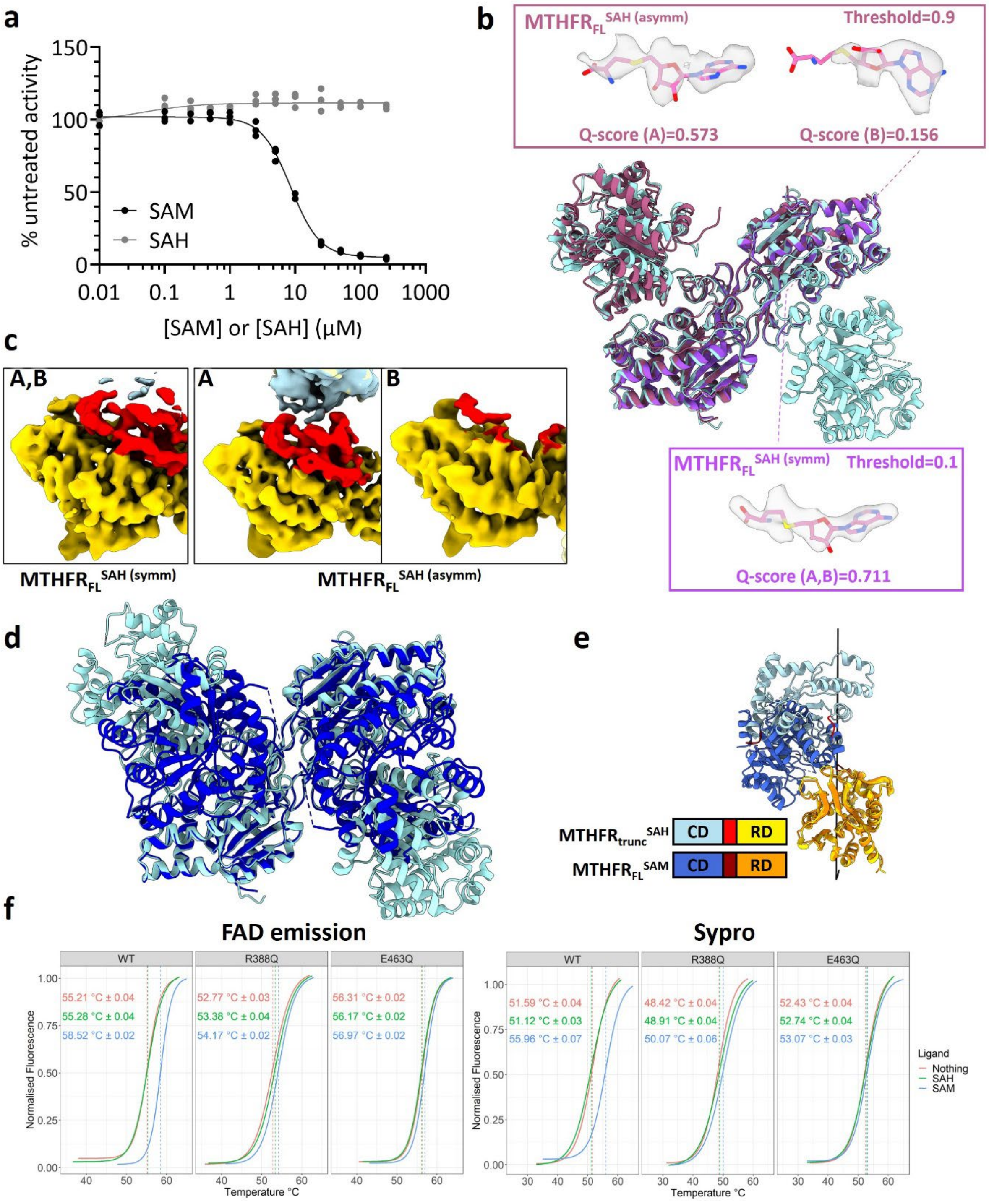

Supplementary Fig. 7 | Structural and biochemical response upon MTHFR$_{FL}$ binding SAM/SAH. a, Relative enzymatic activity of purified recombinant MTHFR$_{FL}$ following incubation with SAM or SAH. N=3 technical replicates. **b**, Cryo-EM models MTHFR$_{FL}^{SAH\ (asymm)}$ (dark pink) (Cα-RMSD 0.826 Å) and MTHFR$_{FL}^{SAH\ (symm)}$ (purple) (C$^{\alpha}$-RMSD 0.536 Å) superimposed on MTHFR$_{trunc}^{SAH}$ (light blue), aligned using the homodimeric RD interface. Inset shows electron densities for the sharpened map at specified threshold and range of 2.2 Å. The average Q-score is also shown for each SAH molecule in their respective protomers (A, B). **c,** Zoomed view of the linker (red) with surrounding regulatory domain (yellow) and catalytic domain (blue) in respective protomer (A, B) of the cryo-EM maps MTHFR$_{FL}^{SAH\ (symm)}$ (left) and MTHFR$_{FL}^{SAH\ (asymm)}$ (middle and right) **d,** Cryo-EM model MTHFR$_{FL}^{SAM}$ (dark blue) (C$^{\alpha}$-

RMSD 0.691 Å) superimposed on the $MTHFR_{trunc}^{SAH}$ crystal structure (light blue). **e**, Hinge axis generated with DynDom[4] comparing the relative position of the catalytic domain between $MTHFR_{FL}^{SAM}$ (dark blue) and $MTHFR_{trunc}^{SAH}$ (light blue). **f**, Differential fluorimetry of FAD emission (left) or Sypro dye (right) of purified recombinant $MTHFR_{FL}$ or protein variants following incubation with 1 mM SAM, 1 mM SAH or no ligand. Tm calculated from inflation point was estimated using a Boltzmann curve fit function in R. N=6 technical replicates. For individual curves and further information see Supplementary Fig. 8a-d.

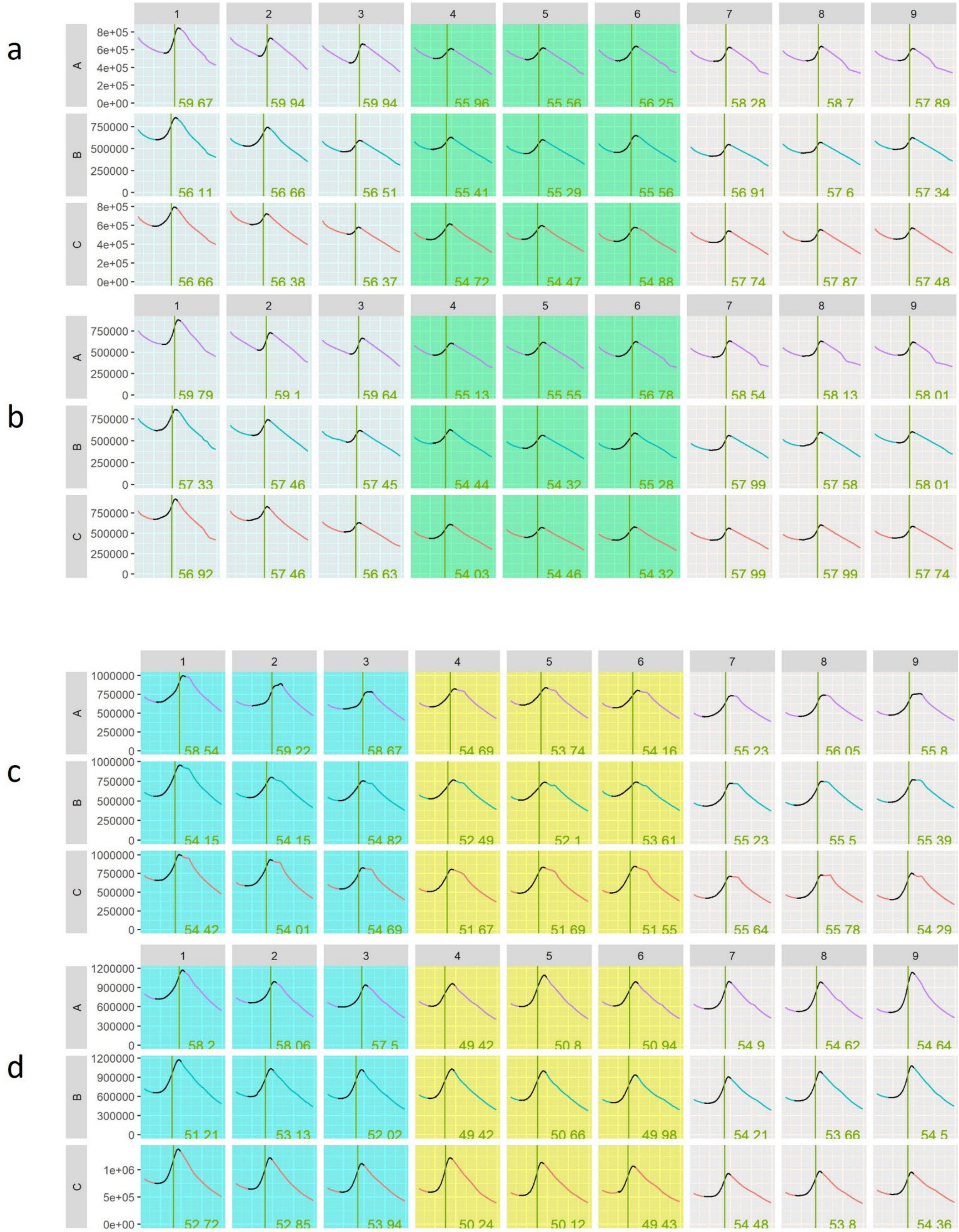

Supplementary Fig. 8 | Raw data for Differential Scanning Fluorimetry. Raw data curves of Fluorescence (y-axis) versus temperature (x-axis) as given from QuantStudio™ 7 Pro and visualised with R. Black curve line represents data which were later normalised and used to derive Tm using

Boltzmann curve fit in R. The temperature range for the black curve was defined by data presenting a positive first derivative surrounding the maximum inclination point (green line and number) as calculated from the raw derivative given by QuantStudio™ 7 Pro. The temperature range was then extended by ±2 degrees. SAM (Rows: A, purple), SAH (Rows: B,blue) and Nothing (Rows: C,red), $MTHFR_{FL}$ (Column 1,2,3), Arg388Gln (Column 4,5,6) and Glu463Gln (Column 7,8,9). Background colour are used to highlight specific MTHFR samples. Each condition was measured with N=6 technical replicates. **a,b** Measurements utilising FAD emission. **c,d** Measurements utilising emission from Sypro orange.

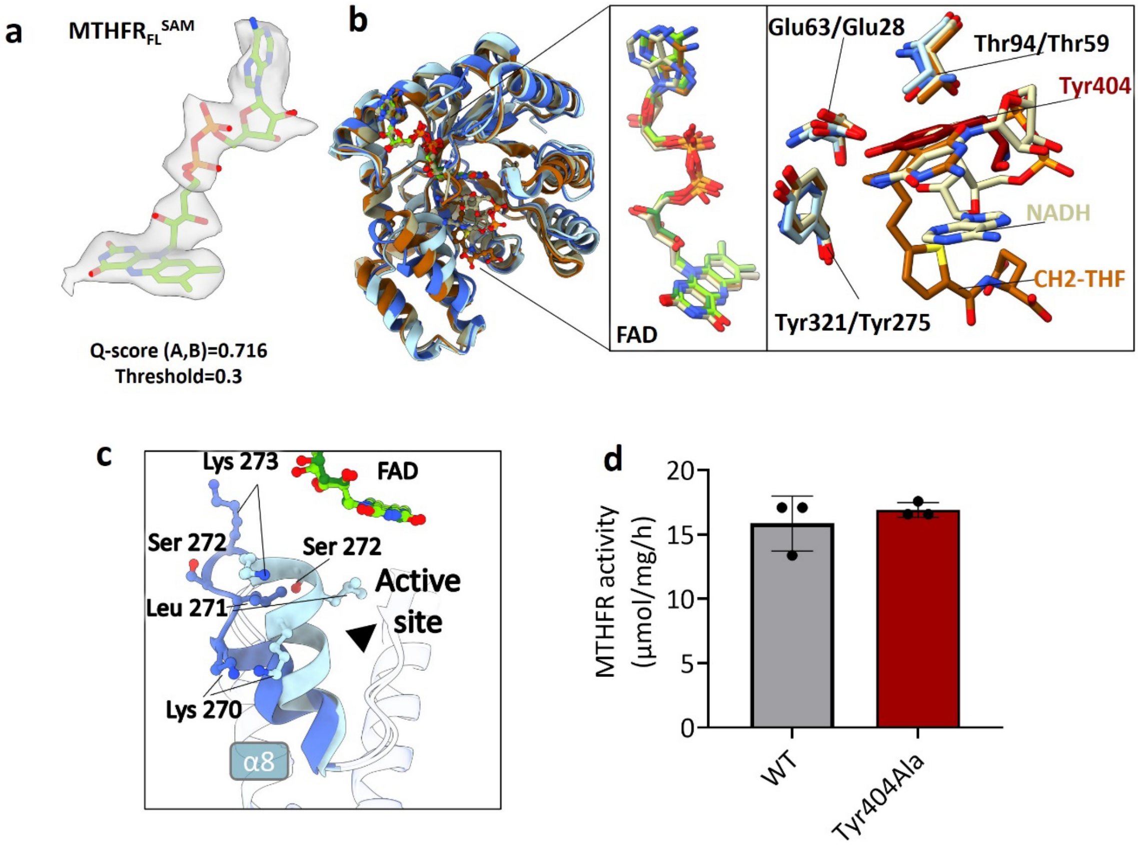

Supplementary Fig. 9 | The catalytic domain accommodates an auto-inhibitory element while maintaining a conserved structural integrity. a, Electron density for the sharpened map at specified threshold, and range of 2.2 Å for FAD found in the respective protomer (A, B) of $MTHFR_{FL}^{SAM}$. The average Q-score is also shown. **b**, Superimposition of human $MTHFR_{trunc}^{SAH}$ (light blue), human $MTHFR_{FL}^{SAM}$ (dark blue), *Escherichia coli* MTHFR co-crystallised with NADH (PDB:1zrq, beige), and *Escherichia coli* MTHFR with Ala177Val background co-crystalized with 5,10-dideazafolate analogue (LY309887) representing the position of 5,10-methylenetetrahydrofolate (CH_2-THF) (PDB:2fmn, brown). Inset showing a zoomed view of FAD (left) and residues interacting with Tyr404 (dark red) within the active site. **c**, Zoomed view of superimposed $MTHFR_{trunc}^{SAH}$ (light blue) and $MTHFR_{FL}^{SAM}$ (dark blue) showing the movement of alpha helix 8 (α8) to accommodate hydrophobic linker insertion in the active site. **d**, Specific activity of overexpressed $MTHFR_{FL}$ (WT) and alanine substitution of Tyr404. N=3 biological replicates. Error bar showing mean ±SD.

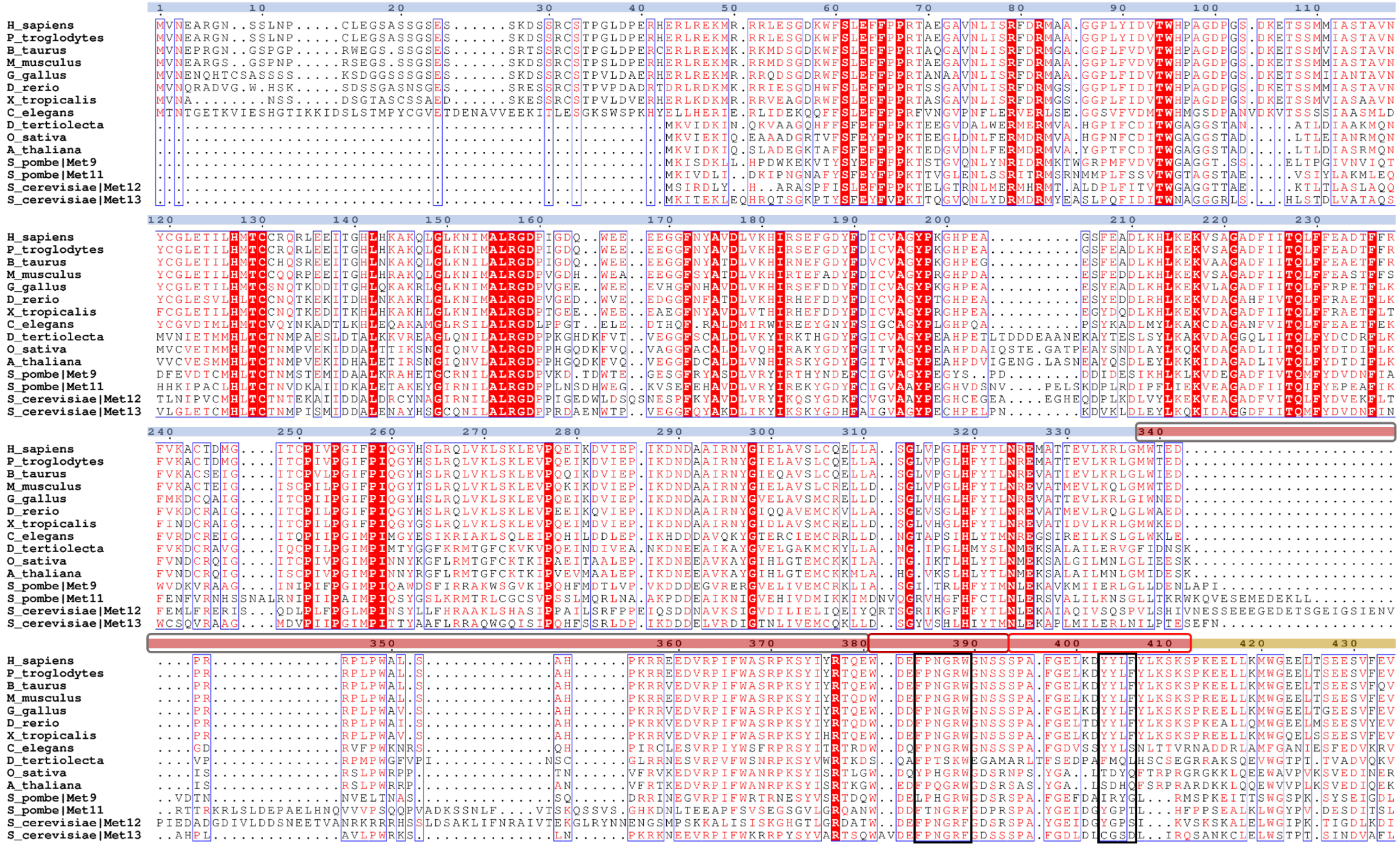
H_sapiens
P_troglodytes
B_taurus
M_musculus
G_gallus
D_rerio
X_tropicalis
C_elegans
D_tertiolecta
O_sativa
A_thaliana
S_pombe|Met9
S_pombe|Met11
S_cerevisiae|Met12
S_cerevisiae|Met13

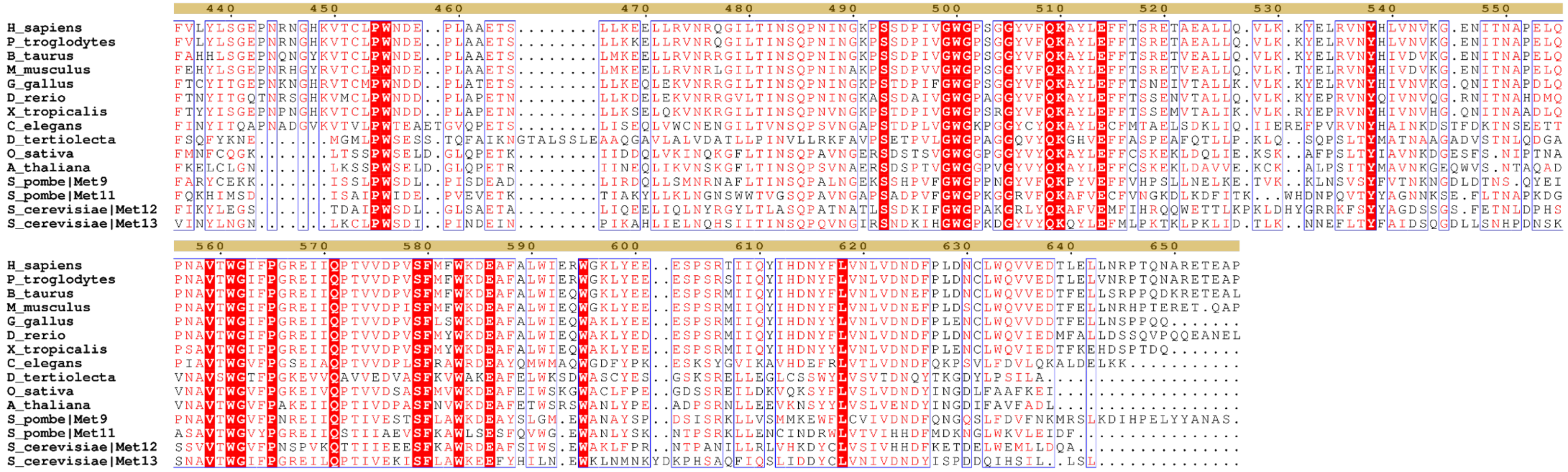

Supplementary Fig. 10 | Sequence alignment for Eucaryotc MTHFR$_{FL}$. Sequences were manually selected from MTHFR entries in UniProt (June 2023) and aligned with Clustal Omega, and visualised with ESPript 3.0 (global score: 0.5) [5]. Marked catalytic domain (1-337), linker (338-412) and regulatory domain (413-656). *Organism*-general name (UniProt ID): *Arabidopsis thaliana* - Mouse-ear cress (O80585), *Bos taurus* - Cattle (Q5I598), *Caenorhabditis elegans* - Nematode worm (Q17693*)*, *Danio rerio* - Zebrafish (B0V153), *Dunaliella tertiolecta* - Green alga (A0A7S3VQX8), *Gallus* - Chicken (E1BXL1), *Homo sapiens* - Human (P42898), *Mus musculus* - House mouse (Q9WU20), *Oryza sativa* - Rice (Q75HE6), *Pan troglodytes* - Chimpanzee (H2PY11), *Saccharomyces cerevisiae* - Brewer's yeast (Met12/P46151, Met13/P53128), *Schizosaccharomyces pombe* - Fission yeast (Met9/Q10258, Met11/O74927), *Xenopus tropicalis* - Western clawed frog (A4IIX8). Sequences features marked: Catalytic domain 1-337 (blue shadow), linker 338-412 (red shadow), regulatory domain 413-656(yellow shadow), LS1 (grey box), LS2 (dark red box), LS3 (orange box), $Y_{403}YLF_{406}$ and $F_{384}PNGRW_{389}$ (black boxes).

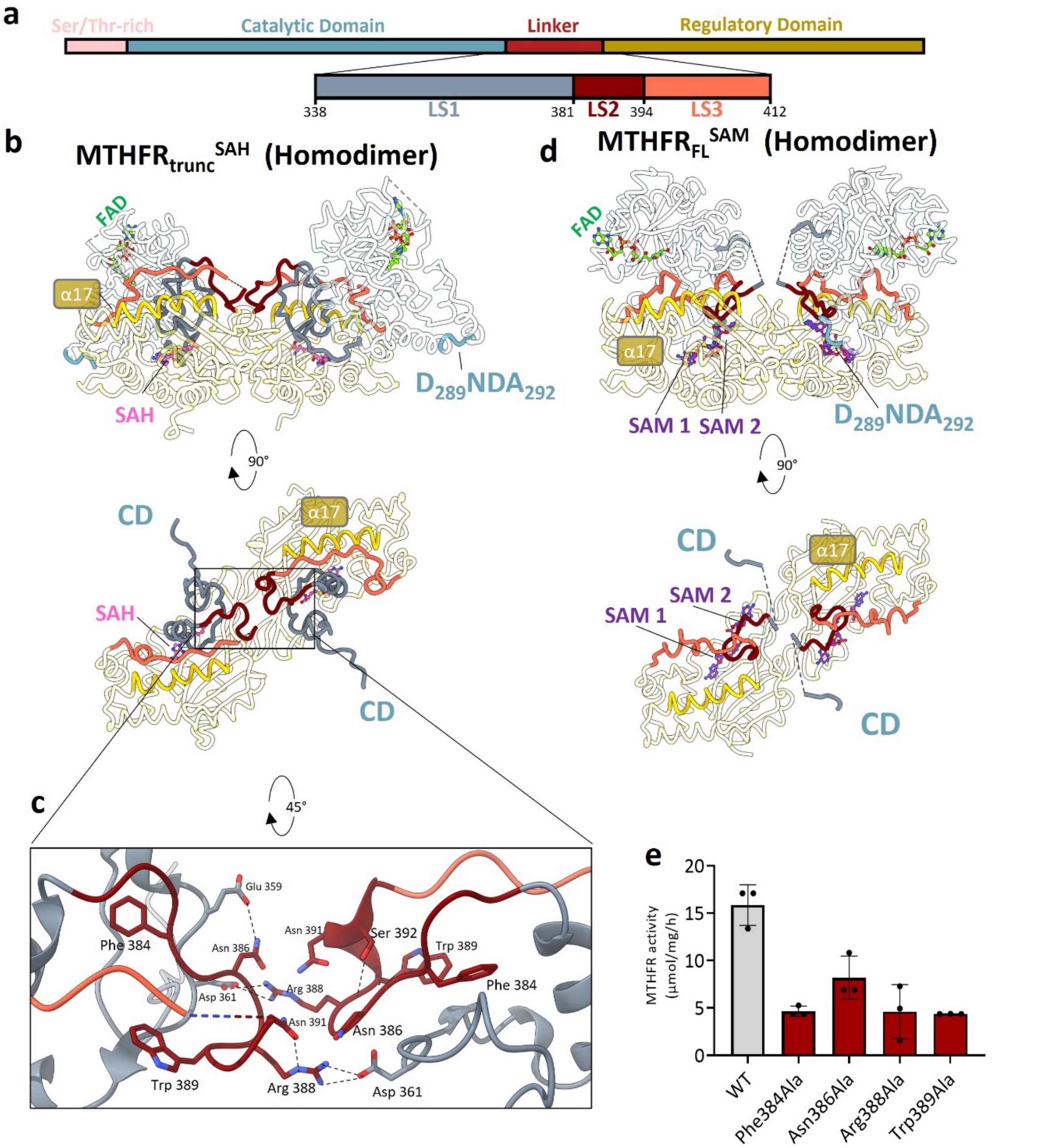

Supplementary Fig. 11 | Rearrangement of linker segments LS1, LS2 and LS3. a, Domain diagram of human $MTHFR_{FL}$, coloured according to MTHFR protein domains and with a zoom-in of linker segments LS1 (aa 338-380), LS2 (aa 381-393) and LS3 (aa 394-412). The same colour coding is maintained in subsequent structural representations **b, c,** and **d.** Transparent models of dis-inhibited $MTHFR_{trunc}^{SAH}$ (**b**) and inhibited $MTHFR_{FL}^{SAM}$ (**d**) with pronounced linker segments LS1, LS2 and LS3, showing their movement in relation to alpha-helix 17 (α17) and CD segment $D_{289}NDA_{292}$. **c**, Inset showing a zoom-in of the homodimeric interface in $MTHFR_{trunc}^{SAH}$, which is further stabilised by interactions between opposing LS2 in the two protomers. **e**, Specific activity data for overexpressed $MTHFR_{FL}$ and alanine substitutions within LS2. N=3 biological replicates. Error bar showing mean ±SD.

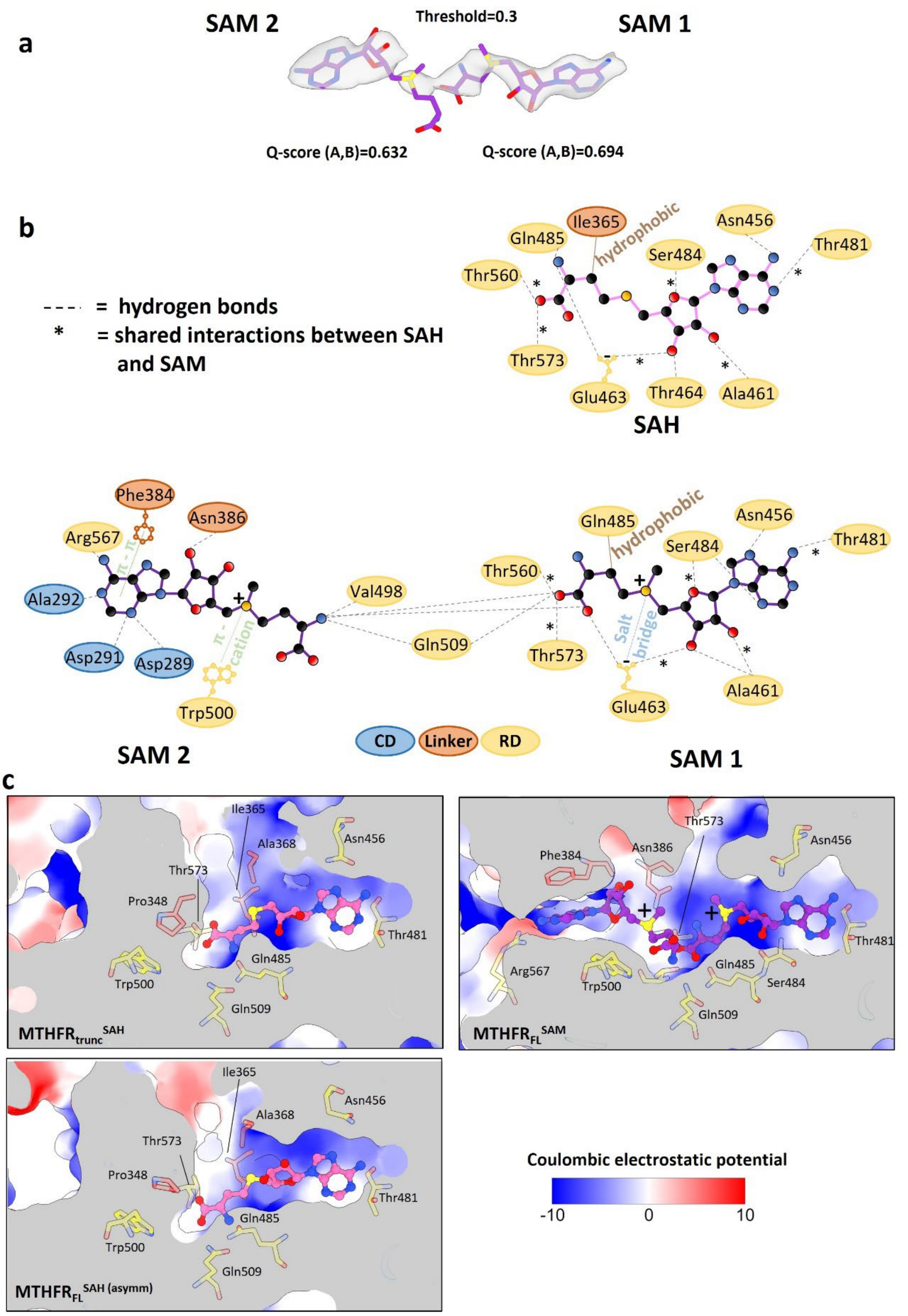

Supplementary Fig. 12 | Structure and properties of the allosteric pocket. a, Electron density for sharpened map at specified threshold and range of 2.2 Å, with Q-score for SAM1 and SAM2 in $MTHFR_{FL}^{SAM}$. **b**, Schematic depicting interactions within the allosteric pocket of $MTHFR_{trunc}^{SAH}$ for SAH,

and within $MTHFR_{FL}^{SAM}$ for SAM1 and SAM2. Indicated interactions were predicted by PLIP[6], except for the hydrogen bond between SAM1 and SAM2, which were predicted by ChimeraX[7].**c**, Allosteric pocket for $MTHFR_{trunc}^{SAH}$, $MTHFR_{FL}^{SAH}$ and $MTHFR_{FL}^{SAM}$ with coulombic charge predicted with ChimeraX. Negative values in blue and positive in red, showing bound SAH (left) or SAM1 and SAM2 (right).

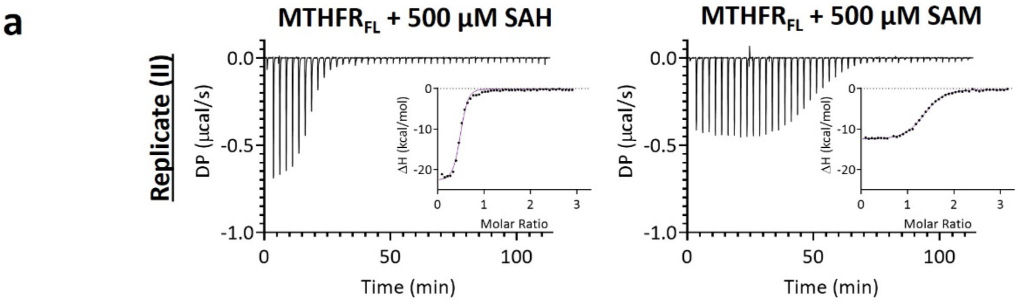

45 injections								
Sample	N ± error	Average ± S.D.	KD ± error (µM)	Average ± S.D.	ΔH ± error (kcal/mol)	Average ± S.D.	-TΔS (kcal/mol)	Average ± S.D.
MTHFR$_{FL}$ + SAM (I)	1.35 ± 0.00	1.33 ± 0.02	0.86 ± 0.04	0.93 ± 0.07	-12.80 ± 0.07	-12.95 ± 0.15	4.48	4.70 ± 0.22
MTHFR$_{FL}$ + SAM (II)	1.31 ± 0.01		0.99 ± 0.08		-13.10 ± 0.13		4.92	
MTHFR$_{FL}$ + SAH (I)	0.48 ± 0.00	0.47 ± 0.01	0.38 ± 0.03	0.38 ± 0.00	-23.70 ± 0.20	-23.40 ± 0.30	15.00	14.65 ± 0.35
MTHFR$_{FL}$ + SAH (II)	0.47 ± 0.00		0.39 ± 0.04		-23.10 ± 0.28		14.30	

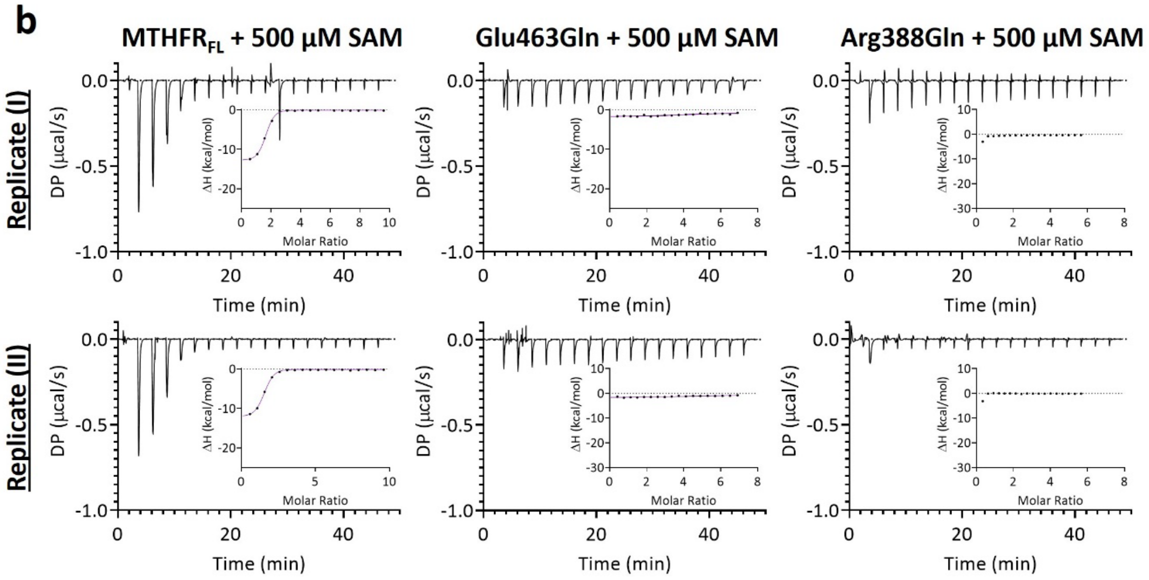

19 injections								
Sample	N ± error	Average ± S.D.	KD ± error (µM)	Average ± S.D.	ΔH ± error (kcal/mol)	Average ± S.D.	-TΔS (kcal/mol)	Average ± S.D.
MTHFR$_{FL}$ + SAM (I)	1.44 ± 0.02	1.40 ± 0.05	0.42 ± 0.07	0.51 ± 0.09	-13.00 ± 0.27	-12.65 ± 0.35	4.33	4.09 ± 0.24
MTHFR$_{FL}$ + SAM (II)	1.35 ± 0.02		0.59 ± 0.07		-12.30 ± 0.22		3.85	
E463Q + SAM (I)	7.67 ± 1.05	7.74 ± 0.07	27.60 ± 32.2	26.90 ± 0.70	-2.13 ± 0.58	-2.08 ± 0.05	-4.09	-4.16 ± 0.07
E463Q + SAM (II)	7.81 ± 0.97		26.20 ± 26.4		-2.02 ± 0.45		-4.23	
R388Q + SAM (I)	N/A	N/A	N/A	N/A	N/A	N/A	N/A	N/A
R388Q + SAM (II)	N/A		N/A		N/A		N/A	N/A

Supplementary Fig. 13 | Isothermal Titration Calorimetry. Isothermal titration calorimetry (ITC) experiments displaying the heat exchange over time. DP: power differential. The insets illustrate ΔH (change in enthalpy) plotted against the molar ratio. All curves have been normalized against buffer injected into respective MTHFR$_{FL}$ variants. **a,** Above: Shows 45 injections of 500 µM SAM or 500 µM SAH into 30 µM purified recombinant MTHFR$_{FL}$. N=2 technical replicates, where replicate I is shown in Fig. 4a. Below: Table of best-fit parameters (**±** corresponding fitting errors) for each replicate and the mean (**±** S.D.) for each sample condition. **b,** Above: Shows 19 injections of 500 µM SAM in 10 µM

purified recombinant $MTHFR_{FL}$ variants. N=2 technical replicates. Below: Table of best-fit parameters (± corresponding fitting errors) for each replicate and the mean (± S.D.) for each sample condition.

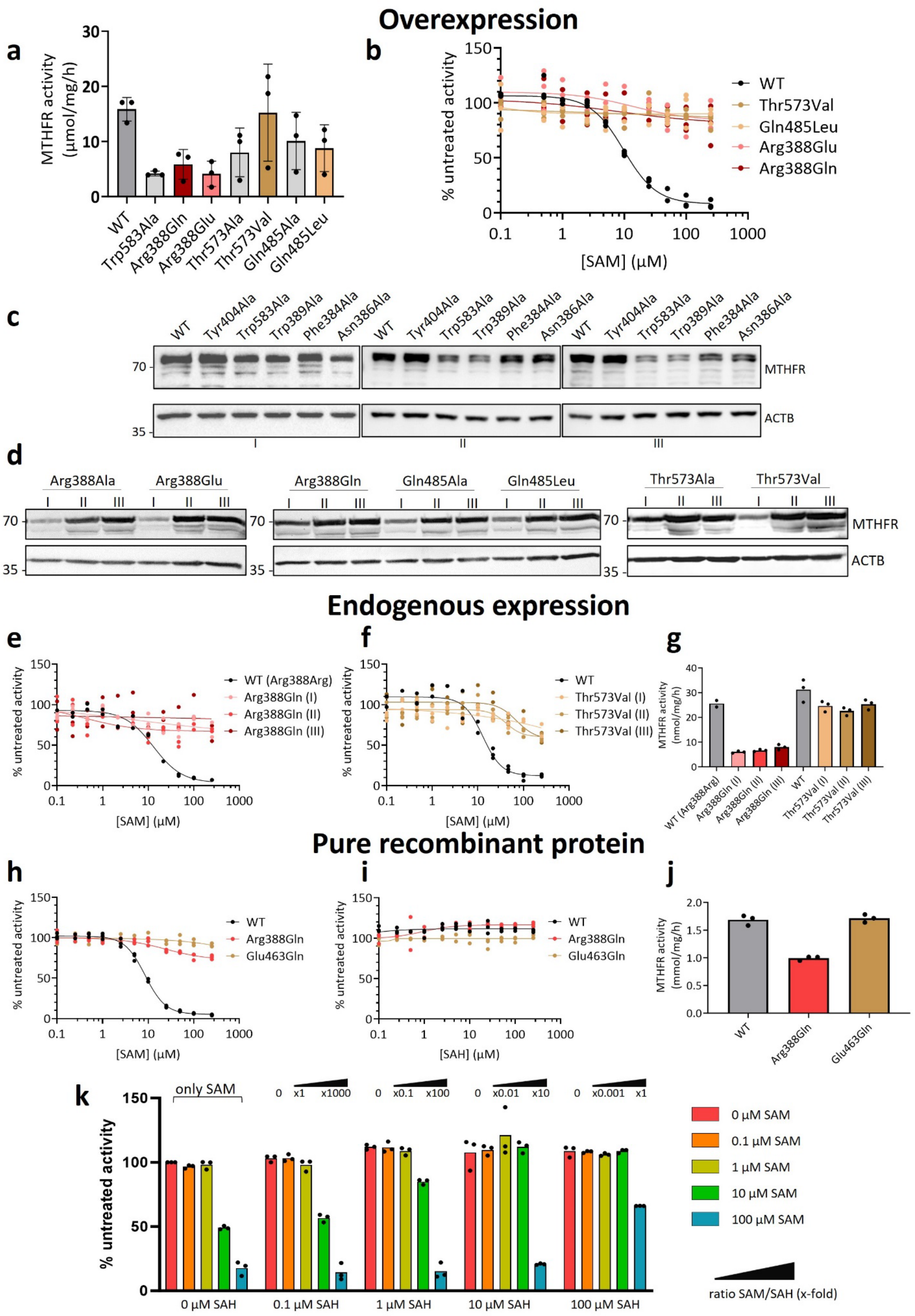

Supplementary Fig. 14 | Activity data for wild-type (WT) MTHFR and variants. Specific activity and

inhibition curves (WT) MTHFR and protein variants overexpressed in MTHFR knock-out 293T cells (**a-d**), genetically engineered for endogenous expression in 293T cells (**e-g**), or *as purified* recombinant $MTHFR_{FL}$ protein following SF9 insect expression (**h-k**). **a**, Specific activity and **b**, Inhibition curves for variants Arg388Gln/Glu, Thr573Val and Gln485Leu with WT corresponding to WT seen in Fig. 2e and Fig. 4b,c. N=3 biological replicates. Error bar showing mean ±SD. Western blotting analysis corresponding to all overexpressed biological protein samples used in Fig. 4b,c and Supplementary Fig. 9d, 11e and 14a-b using **c,** 10% acrylamide gel and **d**, 12% acrylamide gel. Marked band for MTHFR and Anti-β-Actin antibody (ACTB). N=3 biological replicates (I-III). **e-f**, Inhibition curves for endogenously expressed WT MTHFR and three clones each for **e**, Arg388Gln, and **f**, Thr573Val, generated with CRISPR/Cas9 gene editing. **g**, Specific activity for each. WT (Arg388Arg) represents a clone which had undergone CRISPR/Cas9 gene editing but showed no mutation within the MTHFR gene; WT represents the parental, unmodified 293T cells. N=3 technical replicates. **h-j**, Inhibition curves for purified recombinant $MTHFR_{FL}$ (WT) and variants Arg388Gln and Glu463Gln with **h,** SAM and **i**, SAH. **j**, Specific activity. N=3 technical replicates. **k,** Competition assay of purified recombinant $MTHFR_{FL}$ (WT) showing SAM inhibition in the presence of increasing concentration of SAH. Samples were pre-incubated with five different concentrations (0, 0.1, 1, 10 and 100 µM) of SAM and SAH respectively. Indication of SAM/SAH ratio are marked out. N=3 technical replicates.

a

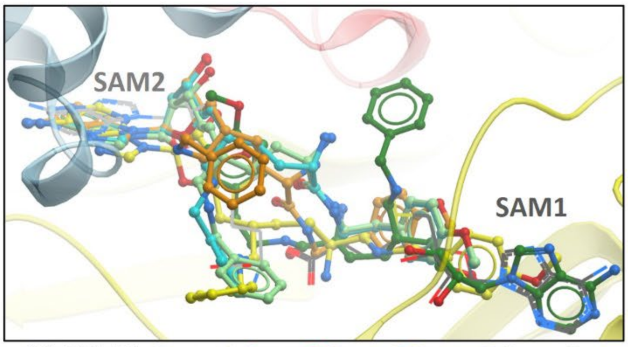

(S)-SKI-72: Pose 1 Pose 2 Pose 3 Pose 4 Pose 5

b

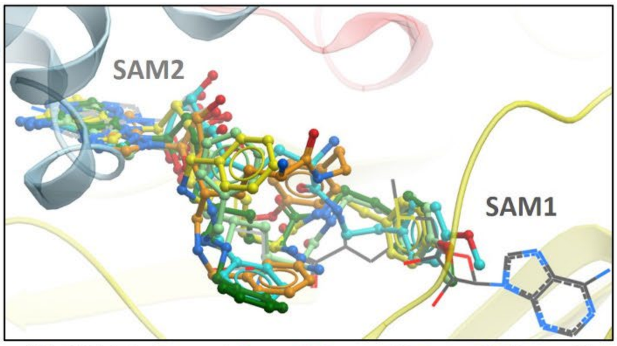

(S)-SKI-72: Pose I Pose II Pose III Pose IV Pose V

c

No.	RTCNN Score	LE Score	ICM-VLS Score	Ligand strain	Van der Waals	Electrostatics	H-bond energy	Hydrophobic energy	Desolvation
**Binding poses starting from SAM1 site (Extended data figure 10k)**									
**1***	-31.07	-10.06	-25.03	14.98	-46.89	25.71	-12.2	-10.14	43.13
**2**	-30.80	-13.29	-21.36	8.07	-50.60	30.99	-11.59	-10.92	46.96
**3**	-30.77	-11.52	-23.27	11.75	-52.31	29.66	-11.08	-10.81	46.04
**4**	-25.47	-3.99	-16.74	12.75	-56.37	37.22	-9.425	-11.35	48.42
**5**	-26.88	-13.76	-28.17	14.41	-45.61	24.60	-14.8	-10.01	46.86
**Binding poses starting from SAM2 site (Extended data figure 10l)**									
**I**	-29.09	-7.18	-18.03	10.85	-50.29	33.15	-10.36	-10.61	44.67
**II**	-25.78	4.25	-6.58	10.83	-51.08	32.74	-6.12	-10.99	47.39
**III**	-27.17	-8.77	-14.68	5.91	-44.74	23.13	-8.49	-9.50	43.26
**IV**	-31.38	-12.96	-19.45	6.49	-50.65	33.12	-11.45	-10.93	47.24
**V***	-26.35	-18.95	-28.73	9.78	-50.39	28.45	-13.34	-10.35	44.50

*Poses shown in Figure 4d

Supplementary Fig. 15 | Docking poses for (S)-SKI-72 docked into MTHFR structure solved in this work. Five poses were obtained each from docking performed after superimposing the adenine ring of (S)-SKI-72 with the equivalent substructure in either **a,** SAM1 or **b,** SAM2. Sticks are colored according to ranking (1 – 5 for panel **a** and I – V for panel **b**) in light green, blue, orange, yellow and dark green respectively. Pose 5 from panel k and pose I from panel l are shown as representative poses in Figure 4d**. c,** Scoring of ICM docking parameters for 5 binding conformations of (S)-SKI-72 starting aligned with SAM1 adenine and 5 binding conformations of (S)-SKI-72 starting aligned with SAM2

adenine. Each row is shaded the same colour as that used for the described binding pose in (**a**) SAM 1 site poses (1-5) or (**b**) SAM 2 site poses (I-V).

Supplementary Table 1 | Cryo-EM data collection, refinement and validation statistics

	MTHFR + SAH symmetric dis-inhibited state (EMDB-18298) (PDB 8QA4)	MTHFR + SAH asymmetric dis-inhibited state (EMDB-18299) (PDB 8QA5)	MTHFR + SAM, inhibited state (EMDB-18300) (PDB 8QA6)
**Data collection and processing**			
Magnification	240,000		240,000
Voltage (kV)	200		200
Electron exposure ($e^{-}/Å^2$)	50		50
Defocus range (μm)	-0.9 to -2.1		-0.9 to -2.1
Pixel size (Å)	0.574		0.574
Symmetry imposed	C2	C1	C2
Initial particle images (no.)	1,980,846	1,980,846	882,089
Final particle images (no.)	105,879	104,101	135,192
Map resolution (Å)	2.8	3.1	2.9
FSC threshold	0.143	0.143	0.143
Map resolution range (Å)	2.5-3.6	2.7-5.4	2.7-5.8
**Refinement**			
Initial model used (PDB code)	6FCX	6FCX	AlphaFold/ SwissProt
Model resolution (Å)	3.0	3.5	3.2
FSC threshold	0.5	0.5	0.5
Map sharpening *B* factor ($Å^2$)	-90.1	-86.3	-105.2
Model composition			
Non-hydrogen atoms	3820	5569	9088
Protein residues	462	814	1106
Ligands	SAH (2)	FAD (1), SAH (2)	FAD (2), SAM (4)
B factors ($Å^2$)			
Protein	29.71	92.83	84.20
Ligand	29.54	224.13	80.55
R.m.s. deviations			
Bond lengths (Å)	0.023	0.027	0.022
Bond angles (°)	2.298	2.318	1.531
Validation			
MolProbity score	1.50	1.48	1.59
Clashscore	3.62	2.37	6.69
Poor rotamers (%)	1.45	1.48	0.52
Ramachandran plot			
Favored (%)	96.49	95.14	96.62
Allowed (%)	3.51	4.74	3.38
Disallowed (%)	0.0	0.12	0.0

Supplementary Table 2 | gRNA and ssODNs used for CRISR/Cas9 homology-directed repair. Parts of the gRNA are highlighted in green, PAM sites in purple, codons of interest are underlined and introduced mutations are displayed in red (desired mutations and PAM site mutations).

	gRNA (5' – 3')	ssODN (5' – 3')
R388Q	ACGAGTTCCCTAACGGCCGC	GTATTTGCAAGGAAGGTCTGCAGGCCCTCACCATTGGCCGTTAGGGAACTCGTCCCACTCCTGGGTACGGTAGATGTAACTCTTTGGTCTGGAGGCCCAGAAGATGGGACGTACATCTTCCTCT
T573V	CTGACGGGATCCACTACGGT	GGCATCTTCCCTGGGCGAGAGATCATCCAGCCCGTCGTAGTGGATCCCGTCAGCTTCATGTTCTGGAAGGTAAAGGAGCCGGGGGCAAGCTTGCCCCGCCCACCTGGAAAACCGTGGGGAGGGA

Supplementary Table 3 | Primers used in site-directed mutagenesis experiments.

Mutation		Primer (5´ --> 3´)
Trp583Ala	F	CCCGTCAGCTTCATGTTCGCGAAGGACGAGGCC
Trp583Ala	R	GGCAAAGGCCTCGTCCTTCGCGAACATGAAGC
Trp389Ala	F	GAGTTCCCTAACGGCCGCGCGGGCAATTCCTC
Trp389Ala	R	AGGGGAAGAGGAATTGCCCGCGCGGCCGTTAGG
Asn386Ala	F	GAGTGGGACGAGTTCCCTGCCGGCCGCTGGG
Asn386Ala	R	GGAATTGCCCCAGCGGCCGGCAGGGAACTCGTC
Phe384Ala	F	ACCCAGGAGTGGGACGAGGCCCCTAACGGCCGC
Phe384Ala	R	GCCCCAGCGGCCGTTAGGGGCCTCGTCCCACTC
Tyr404Ala	F	GGGGAGCTGAAGGACTACGCCCTCTTCTACCTG
Tyr404Ala	R	GCTCTTCAGGTAGAAGAGGGCGTAGTCCTTCAG
Thr573Ala	F	CGAGAGATCATCCAGCCCGCCGTAGTGGATCCC
Thr573Ala	R	GCTGACGGGATCCACTACGGCGGGCTGGATGAT
Thr573Val	F	CGAGAGATCATCCAGCCCGTCGTAGTGGATCCC
Thr573Val	R	GCTGACGGGATCCACTACGACGGGCTGGATGAT
Glu463Gln	F	GATGAGCCCCTGGCGGCTCAGACCAGCCTGCTG
Glu463Gln	R	CTCCTTCAGCAGGCTGGTCTGAGCCGCCAGGGG
Arg388Ala	F	GACGAGTTCCCTAACGGCGCCTGGGGCAATTCC
Arg388Ala	R	GGAAGAGGAATTGCCCCAGGCGCCGTTAGGGAA
Arg388Gln	F	GACGAGTTCCCTAACGGCCAATGGGGCAATTCC
Arg388Gln	R	GGAAGAGGAATTGCCCCATTGGCCGTTAGGGAA
Arg388Glu	F	GACGAGTTCCCTAACGGCGAATGGGGCAATTCC
Arg388Glu	R	GGAAGAGGAATTGCCCCATTCGCCGTTAGGGAA
Gln485Ala	F	ATCCTCACCATCAACTCAGCGCCCAACATCAAC
Gln485Ala	R	CTTCCCGTTGATGTTGGGCGCTGAGTTGATGGT
Gln485Leu	F	ATCCTCACCATCAACTCACTGCCCAACATCAAC
Gln485Leu	R	CTTCCCGTTGATGTTGGGCAGTGAGTTGATGGT

Supplementary References

1. Froese, D. S. *et al.* Structural basis for the regulation of human 5,10-methylenetetrahydrofolate reductase by phosphorylation and S-adenosylmethionine inhibition. *Nature Communications 2018 9:1* **9**, 1–13 (2018).

2. Afonine, P. V. *et al.* Real-space refinement in PHENIX for cryo-EM and crystallography. *Acta Crystallogr D Struct Biol* **74**, 531–544 (2018).

3. Pintilie, G. *et al.* Measurement of atom resolvability in cryo-EM maps with Q-scores. *Nature Methods 2020 17:3* **17**, 328–334 (2020).

4. Hayward, S. & Berendsen, H. J. C. Systematic Analysis of Domain Motions in Proteins From Conformational Change: New Results on Citrate Synthase and T4 Lysozyme. *Proteins* **30**, 144–154 (1998).

5. Robert, X. & Gouet, P. Deciphering key features in protein structures with the new ENDscript server. *Nucleic Acids Res* **42**, W320–W324 (2014).

6. Adasme, M. F. *et al.* PLIP 2021: expanding the scope of the protein-ligand interaction profiler to DNA and RNA. *Nucleic Acids Res* **49**, W530–W534 (2021).

7. Pettersen, E. F. *et al.* UCSF ChimeraX: Structure visualization for researchers, educators, and developers. *Protein Sci* **30**, 70–82 (2021).
