# Supplementary material for: Dynamic inter-domain transformations mediate the allosteric regulation of human 5, 10-methylenetetrahydrofolate reductase
Source: Nat Commun. 2024 Apr 15;15:3248. doi: 10.1038/s41467-024-47174-y (PMC11018872; doi:10.1038/s41467-024-47174-y)
Supplement: Supplementary file 3 — Reporting Summary [file 41467_2024_47174_MOESM3_ESM.pdf]

nature portfolio

Corresponding author(s): Wyatt W. Yue, D. Sean Froese, or Thomas J. McCorvie

Last updated by author(s): 15/03/2024

Reporting Summary

Nature Portfolio wishes to improve the reproducibility of the work that we publish. This form provides structure for consistency and transparency in reporting. For further information on Nature Portfolio policies, see our Editorial Policies and the Editorial Policy Checklist.

Statistics

For all statistical analyses, confirm that the following items are present in the figure legend, table legend, main text, or Methods section.

n/a	Confirmed	
☐	☒	The exact sample size (*n*) for each experimental group/condition, given as a discrete number and unit of measurement
☐	☒	A statement on whether measurements were taken from distinct samples or whether the same sample was measured repeatedly
☒	☐	The statistical test(s) used AND whether they are one- or two-sided<br>*Only common tests should be described solely by name; describe more complex techniques in the Methods section.*
☒	☐	A description of all covariates tested
☒	☐	A description of any assumptions or corrections, such as tests of normality and adjustment for multiple comparisons
☐	☒	A full description of the statistical parameters including central tendency (e.g. means) or other basic estimates (e.g. regression coefficient) AND variation (e.g. standard deviation) or associated estimates of uncertainty (e.g. confidence intervals)
☒	☐	For null hypothesis testing, the test statistic (e.g. *F*, *t*, *r*) with confidence intervals, effect sizes, degrees of freedom and *P* value noted<br>*Give P values as exact values whenever suitable.*
☒	☐	For Bayesian analysis, information on the choice of priors and Markov chain Monte Carlo settings
☒	☐	For hierarchical and complex designs, identification of the appropriate level for tests and full reporting of outcomes
☒	☐	Estimates of effect sizes (e.g. Cohen's *d*, Pearson's *r*), indicating how they were calculated

Our web collection on statistics for biologists contains articles on many of the points above.

Software and code

Policy information about availability of computer code

Data collection	York Structural Biology Laboratory (YSBL) Glacios equipped with a Falcon 4 direct electron detector (Thermo Fisher Scientific), Microscopy Research Services (EMRS) facility at Newcastle University Hitachi HT7800 microscope, QuantStudio 5 and 7 Pro (Applied Biosystems), HPLC (Jasco), ChemiDoc Touch Imaging System (Bio-Rad), MicroCal PEAQ-ITC microcalorimeter (Malvern Instruments, Malvern, UK)
Data analysis	CryoSPARC-v4.2.1, GraphPad Prism v9, COOT v0.9.8.1, SWISS-MODEL, UCSF ChimeraX v1.5, PHENIX v1.20.1-4487, ICM-Pro software , ISOLDE, AlphaFold v2.0, Namdinator, R v4.2.0, Chemdraw v22.2.0, MicroCal PEAQ-ITC software

For manuscripts utilizing custom algorithms or software that are central to the research but not yet described in published literature, software must be made available to editors and reviewers. We strongly encourage code deposition in a community repository (e.g. GitHub). See the Nature Portfolio guidelines for submitting code & software for further information.

Data

Policy information about availability of data

All manuscripts must include a data availability statement. This statement should provide the following information, where applicable:

- Accession codes, unique identifiers, or web links for publicly available datasets
- A description of any restrictions on data availability
- For clinical datasets or third party data, please ensure that the statement adheres to our policy

Structures and EM maps have been deposited in the Protein Data Bank (PDB) and Electron Microscopy Data Resource (EMD) under the accession codes of

PDB-8QA4 [http://doi.org/10.2210/pdb8QA4/pdb] /EMDB-18298 [https://www.ebi.ac.uk/pdbe/entry/emdb/EMD-18298] (MTHFR + SAH symmetric dis-inhibited state),
PDB-8QA5 [http://doi.org/10.2210/pdb8QA5/pdb]/EMDB-18299 [https://www.ebi.ac.uk/pdbe/entry/emdb/EMD-18299] (MTHFR + SAH asymmetric dis-inhibited state) and PDB-8QA6 [http://doi.org/10.2210/pdb8QA6/pdb]/EMDB-18300 [https://www.ebi.ac.uk/pdbe/entry/emdb/EMD-18300] (MTHFR + SAM, inhibited state). Cryo-EM data have been deposited to the Electron Microscopy Public Image Archive (EMPIAR), EMPIAR-11959 [https://www.ebi.ac.uk/empiar/EMPIAR-11959] (MTHFR + SAH) and EMPIAR- 11926 [https://www.ebi.ac.uk/empiar/EMPIAR-11926](MTHFR + SAM).
Structure not generated in this study, PDB-6FCX [http://doi.org/10.2210/pdb6FCX/pdb] PDB-1ZRQ [http://doi.org/10.2210/pdb1ZRQ/pdb] and PDB-2FMN [http://doi.org/10.2210/pdb2FMN/pdb]. All main data supporting the findings of this study are available within the article and Supplementary Information. Source data are provided with this paper.

Research involving human participants, their data, or biological material

Policy information about studies with human participants or human data. See also policy information about sex, gender (identity/presentation), and sexual orientation and race, ethnicity and racism.

Reporting on sex and gender	n/a
Reporting on race, ethnicity, or other socially relevant groupings	n/a
Population characteristics	n/a
Recruitment	n/a
Ethics oversight	n/a

Note that full information on the approval of the study protocol must also be provided in the manuscript.

Field-specific reporting

Please select the one below that is the best fit for your research. If you are not sure, read the appropriate sections before making your selection.

☒ Life sciences ☐ Behavioural & social sciences ☐ Ecological, evolutionary & environmental sciences

For a reference copy of the document with all sections, see nature.com/documents/nr-reporting-summary-flat.pdf

Life sciences study design

All studies must disclose on these points even when the disclosure is negative.

Sample size	For Cryo-EM structural determination 5606 micrographs and 2394 micrographs were respectively collected, and sample size were determined based on the adequate resolution given by the data.<br><br>For functional assays, no statistical methods were used to determine sample size, but rather based on commonly used sample sizes in the filed (generally 2-3 repeats). Each technical replicate, alternatively biological replicates, are shown as individual points. In DSF, where N=6 technical replicates were pooled (individual curves found in supplementary), the size was chose based on data availability of two separate runs (using same source of protein and reagents) each with three technical replicates.
Data exclusions	No data was systematically excluded. Single data points in the functional assays that were excluded due to obvious error in sample handling are clearly marked within the source data.
Replication	Activity measurements were carried out in technical replicates or biological replicates of N=3 or 6 as stated in figure legends. ITC were carried out in technical replicates N=2. All attempts were successful, no other attempts beside what is shown in the article were made with the same experimental set-up.
Randomization	Not applicable - no experimental groups were involved.
Blinding	Not applicable - no group allocation was involved.

Reporting for specific materials, systems and methods

We require information from authors about some types of materials, experimental systems and methods used in many studies. Here, indicate whether each material, system or method listed is relevant to your study. If you are not sure if a list item applies to your research, read the appropriate section before selecting a response.

Materials & experimental systems

n/a	Involved in the study
	☒ Antibodies
	☒ Eukaryotic cell lines
☒	Palaeontology and archaeology
☒	Animals and other organisms
☒	Clinical data
☒	Dual use research of concern
☒	Plants

Methods

n/a	Involved in the study
☒	ChIP-seq
☒	Flow cytometry
☒	MRI-based neuroimaging

Antibodies

Antibodies used: monoclonal mouse anti-flag (Sigma, F3165), monoclonal mouse anti-ACTB (sigma, A1978), mouse IgG kappa binding protein conjugated to Horseradish Peroxidase (Santa Cruz, sc-516102)

Validation: All antibodies were validated by the vendor, and reports can be found on the vendors websites.
(Sigma, F3165): https://www.sigmaaldrich.com/CH/en/product/sigma/f3165
(Sigma, A1978): https://www.sigmaaldrich.com/CH/en/product/sigma/a1978?utm_source=google&utm_medium=cpc&utm_campaign=18067744937&utm_content=140940134115&gclid=Cj0KCQjwwMqvBhCtARIsAIXsZpYv8sE0SIxBk0DsPOzrDRqbLF97HSlKY9LfEGRZzFqFigS46XGr-p4aAmfeEALw_wcB&icid=sharepdp-clipboard-copy-productdetailpage
Santa Cruz, sc-516102): https://www.scbt.com/p/m-igg-kappa-bp-hrp

Eukaryotic cell lines

Policy information about cell lines and Sex and Gender in Research

Cell line source(s): (Spodoptera frugiperda: SF9 cells (#12659017, Gibco) further product information https://www.atcc.org/products/crl-3216 , HEK293T (CRL-3216, ATCC) further product information https://www.thermofisher.com/order/catalog/product/12659017

Authentication: No cells lines used were authenticated by us.

Mycoplasma contamination: Cell lines were not tested for mycoplasma contamination

Commonly misidentified lines (See ICLAC register): No commonly misidentified lines were used in this study.

Plants

Seed stocks: n/a

Novel plant genotypes: n/a

Authentication: n/a
